# Consumer Consent Regulation

**DOI:** 10.1515/ger-2024-0124

**Published:** 2025-06-12

**Authors:** Roland Strausz

**Affiliations:** Berlin School of Economics, 9373 Humboldt-Universität zu Berlin, Berlin, Germany

**Keywords:** privacy regulation, data collection, price discrimination, L51, D82

## Abstract

Consumer consent regulation is the cornerstone of modern data privacy regulation such as the European GDPR and the Californian CCPA. By ensuring that consumers can reject any harmful data collection, the regulation seems an effective tool for protecting consumers against price discrimination. By contrast, I provide the insight that consent regulation alone is ineffective because it provides firms with the loophole to commit to unattractive offers to dissenting consumers. Effective consent regulation therefore requires an explicit regulation of the firm’s dissent offer. This is informationally demanding; regulation that merely insists on “reasonable” (sequential rational) offers is ineffective.

## 1 Introduction

The current progress in data collection and processing holds the potential to significantly boost economic prosperity. Many consumers nowadays benefit from personalized recommendations for movies, books, or other products as firms learn consumers personal preferences through data collection. More significantly, information about consumers’ personal preferences is a crucial driver of innovation because it allows firms to create new products that align more closely with consumer preferences. Thus, consumers, firms, and society as a whole stand to benefit substantially from the digitalization of consumer markets with its advanced data collection abilities.

However, in markets with imperfect competition data collection can also harm consumers, particularly when it enables a firm to discern a consumer’s willingness to pay. The firm then benefits from exercising its market power by charging a high personalized price to those consumers who value the product most. These consumers lose from the data collection as they end up paying higher prices than without the data collection. In general, the overall impact of such price discrimination on consumer surplus and aggregate surplus is however complex and contingent on the specifics of the data collection. As Bergemann et al. (2015) show, price discrimination in general allows a large range of feasible economic outcomes with highly ambiguous effects on consumers.

Given the ambiguous effects of data collection and the limited information available to regulators, a market-driven, indirect approach has been adopted: *consumer consent regulation*. This regulation requires firms to obtain explicit consent from consumers or to give consumers an explicit opt-out right. The regulation effectively grants consumers ownership rights over their information, thereby creating a market for data collection. Thus, in the spirit of Coase (1960), the regulation aims to leverage the power of market forces to obtain efficient outcomes. Prominent examples of requiring explicit consent include the European Union’s General Data Protection Regulation (GDPR) and its Digital Markets Act (DMA). Similarly, the California Consumer Privacy Act (CCPA) enables consumers to reject any harmful data collection, by demanding firms to provide an explicit opt-out right.1Hence, while the European and Californian exhibit a different status quo of when the consumer is inactive, both approaches empower the consumer to give or withhold her consent.


The rationale behind such consent regulation is straightforward: Just as a firm will not engage in data collection that hurts its profits, a consumer will prevent any data collection that hurts him as a consumer. This reasoning suggests that data collection that occurs with the consumer’s consent must benefit both parties and, thus, increases aggregate surplus. Hence, consent regulation seems a panacea, ensuring data collection to the benefit of all.

This paper provides the insight that the above reasoning is incomplete as it fails to account for the firm’s offer contingent on the consumer’s refusal to consent, the firm’s dissent offer. The implicit but erroneous assumption behind the reasoning is that by refusing data collection, the dissenting consumer receives the offer that would obtain without any data collection possibilities. This paper argues that, to the firm, such an offer is suboptimal. By contrast, the firm is, in general, strictly better of committing to a dissent offer that is worse to the consumer than if data collection were not possible. This prevents consent regulation to protect consumers against harmful price discrimination.

Regulations such as the GDPR and the CCPA allow such contingent offers by design. The GDPR does so because by explicitly requiring a firm to clarify to the consumer how it uses the data, the regulation also provides the firm with the ability to state what happens without consent.2In practice, this is done in the form of pop-up messages when a new consumer accesses a firm’s website. These pop-up messages not only ask for the explicit consent of the consumer, but also carefully explain the implications of the consumer’s choice. Appealing to the concept of reasonableness, the CCPA is even more explicit in this regard. For instance, in article CIV 1798.125.2, it explicitly clarifies: “Nothing in this subdivision prohibits a business from charging a consumer a different price or rate, or from providing a different level or quality of goods or services to the consumer, if that difference is reasonably related to the value provided to the business by the consumer’s data.”3See CIV 1798.125.2, last retrieved 2024.11.07. The CCPA therefore explicitly allows the firm to commit to dissent offers that differ from a consent offer but with the requirement that they are “reasonable”.

Hence, while it is correct that the consumer consent model gives consumers the ability to decline data collection if it is not in their interests, such regulations also enable firms to commit to a dissent offer. Without further restrictions, this commitment provides firms with a simple loophole, circumventing the regulation’s ability to protect consumers. Firms just have to commit to a dissent offer that is so unattractive that the consumer is better off consenting. It is therefore imperative to flank consumer consent regulation with additional measures to prevent such exploitation of this loophole. This paper studies the effectiveness of such additional measures.

Indeed, by ensuring that consent is not coerced, Article 7.4 of the GDPR indicates that EU regulators are, in principle, aware of this loophole: “When assessing whether consent is freely given, utmost account shall be taken of whether, *inter alia*, the performance of a contract, including the provision of a service, is conditional on consent to the processing of personal data that is not necessary for the performance of that contract.”4See Article 7, last retrieved 2024.11.07. However, the article’s formulation of “utmost account” allows much room for interpretation. Indeed, as of yet no regulatory actions or court cases explicitly refer to Article 7.4, even though some regulatory actions can be attributed to its underlying principles. Likewise, the CCPA’s “right to no discrimination,” which stipulates that consumers cannot be discriminated against for exercising their privacy rights, indicates a similar awareness.5E.g., Bloomberg Law last retrieved 2024.11.07.


From a pure theoretical perspective, the loophole is easily fixed. One simply extends consent regulation by the requirement that firms must provide to dissenting consumers the offer that mirrors the outcome that would exist without the possibility of data collection. This extension requires however that regulators understand the counterfactual scenario of a world without data collection. Such regulation may therefore be too informational demanding.

Because of this drawback, I evaluate the efficacy of more pragmatic regulatory approaches for regulating the alternative available to consumers who refuse consent. In particular and in line with CCPA’s CIV 1798.125.2 as quoted above, I investigate regulation that mandates firms to extend only “reasonable” offers to consumers.

Considering a “reasonable offer” as one that is sequential rational to the firm, I show that such extended regulation is however also ineffective.6See Inazu (2020) or Jaeger (2023), for discussions concerning the concept of “reasonableness” in the rule of law. This is so because data collection makes private information verifiable. Indeed, because private information that is verifiable tends to unravel and induces voluntary disclosure, sequential rational offers actually also undermine consent regulation.7For economic literature on verifiable private information inducing voluntary disclosure see, for example, Grossman (1981) and Milgrom (1981). In particular, the offer that a consumer receives without any possibility of data collection (and, as previously argued, would extend consent regulation into an effective regulation), is not sequential rational.

Concerning the related literature, closest related is Hermalin and Katz (2006), who analyze the role of data property rights on equilibrium outcomes in both competitive and monopolistic settings. While not addressing regulatory measures beyond pure data property rights, they show that if firms can commit to offers before consumers decide on a verifiable disclosure of their personally identifiable data, then the set of equilibrium outcomes is independent of whether the firms or the consumers possess the property rights to the data. This result is inline with the insight above that, because data collection makes private information verifiable, Coase (1960)’s idea to complete the market by defining property rights does not resolve data collection issues.

Hermalin and Katz (2006) moreover derive conditions such that their irrelevance result also obtains when firms cannot commit to offers before the consumers’ disclosure decision. Focusing on this non-commitment case, Ali et al. (2023a, 2023b) expand on this result by showing that, depending on the type of disclosure, the unraveling effects of verifiable private information may undermine consent regulation and also note that a small concealment cost reinforces unraveling. Also the law and economics literature points to the problem of unravelling as undermining a property right approach towards privacy (e.g., Posner 1998; Pepper 2011).

In line with Ali et al. (2023b) but focusing on the seller’s commitment, the insights of this paper also offer a simple, alternative explanation for the empirically observed “privacy paradox” of consumers, whose self-reported high valuation for data privacy stand in conflict with their frequent willingness to exchange data for only minimal compensation (e.g., Berendt et al. 2005; Athey et al. 2017). The explanation is that, even if consumers much value their data privacy, a firm does not need to offer *any* compensation to such consumers when it makes the offer for dissenting consumers unattractive enough. This explanation offers an alternative to theories that emphasize a social externality of data collection (e.g., Choi et al. 2019; Ichihashi 2021; Acemoglu et al. 2022; Bergemann, Bonatti, and Gan 2022) or experimental studies that study the bounded rationality of consumers (e.g., Beresford et al. 2012; Athey et al. 2017).

The remainder of the paper formally derives the results as discussed above by analyzing a straightforward but canonical model of a seller fully learning the buyers’ willingness to pay through data collection.

## 2 Model and Analysis

### 2.1 Model

Consider a firm producing an indivisible good for consumers at a normalized cost of zero. A specific consumer values the good at some valuation *v* ∈ [0, 1]. Initially, each consumer is privately informed about her personal valuation so that, without the consumer’s data, the firm only knows that the consumer’s value is distributed over [0,1] with a cumulative distribution function *F*(*v*) that admits a continuous, strictly positive density *f*(*v*) > 0.

By contrast, the firm can learn (or verify) the valuation of a particular consumer by collecting and processing her data. The collection and processing of consumer data is costless to the firm and allows the firm to learn the consumer’s valuation *v* perfectly.

The modelling assumption that data collection allows the firm to perfectly learn the consumer’s type is not crucial. It however illustrates the case in which the trade-off between the welfare benefits of data collection and its potential threat to consumers is extreme. In particular, when the firm perfectly learns the consumer’s type, the data collection, as explicitly shown below, allows the firm to extract all consumer rents.8If, by contrast, the firm’s learning were imperfect, consumer surplus would not be zero with the data collection, but, adopting the notation as introduced below, rather some $\hat{CS^{d}}\geq 0$ and the firm would earn some profit, ${\hat{\Pi }}^{d}$ , where the pair $\left(\hat{CS^{d}},{\hat{\Pi }}^{d}\right)$ lies in the “feasibility triangle” as derived in Bergemann et al. (2015). All results then hold with respect to the profit levels ${\hat{\Pi }}^{d}$ and consumer surplus levels $\hat{CS^{d}}$ rather than the specific values of Π^*d*^ and *CS* ^*d*^ = 0 as computed below.


### 2.2 No Data Collection (Benchmark)

As the research question of this paper is to understand the extent to which consumers, firms, and society as a whole may benefit from the digitalization of consumer markets with its advanced data collection abilities, the appropriate benchmark for my analysis is a situation in which the market interaction takes place without any data collection. Subsequently, I analyze potential Pareto improvements vis-a-vis this benchmark.

In the absence of any data collection, the firms expects a demand *D*(*p*) = 1 − *F*(*p*) when setting a price *p*.9
Riley and Zeckhauser (1983) show that a take-it-or-leave-it offer in the form of a single price is profit-maximizing. Hence, the firm’s optimal price solves

$$\underset{p}{\max}\Pi \left(p\right)=p\left(1-F\left(p\right)\right).$$

Based on the first order condition, the firm’s optimal price, *p* ^*n*^, is implicitly defined by

(1) $$p^{n}=\frac{1-F\left(p^{n}\right)}{f\left(p^{n}\right)}.$$

and, hence, satisfies *p* ^*n*^ ∈ (0, 1).10Existence of *p* ^*n*^ ∈ (0, 1) follows from the intermediate value theorem: By the continuity of *f*(.), both the LHS and RHS of the equality (1) are continuous. For *p* = 0, the LHS, 0, is smaller than the RHS, 1/*f*(0) > 0. For *p* = 1, the LHS, 1, exceeds the RHS, 0. The intermediate value theorem therefore implies there is a *p* ^*n*^ ∈ (0, 1) such that (1) holds. Consequently, the firm’s optimal profits are

$$\Pi^{n}\equiv p^{n}\left(1-F\left(p^{n}\right)\right)$$

and consumer surplus is

$$CS^{n}\equiv \int_{p^{n}}^{1}\left[v-p^{n}\right]dF\left(v\right)>0.$$

The analysis confirms that, in the sense of Galbraith (1952), the consumer’s private information acts as a countervailing power against the firm’s market power. The consumer’s private information prevents the monopolist from extracting the full surplus, resulting in a positive consumer surplus in the form of information rents.

Although a positive consumer rent is a common outcome in models of monopolistic screening, the analysis below shows that when data collection makes private information verifiable, private information alone is insufficient to generate consumer rents, even under consent regulation.

### 2.3 Data Collection Without Consent

Next suppose the market interaction takes place in a digital world with data collection but one in which the firm does not need the consumer’s consent to learn the consumer’s valuation through its data collection.

In this case, it is clearly optimal for the firm to collect the consumer’s data, thereby learning her specific valuation *v*, and then charging a price that corresponds to the learned valuation. This yields the firm an expected profit of

$$\Pi^{d}\equiv \int_{0}^{1}vdF\left(v\right)>\Pi^{n}.$$

As the firm extracts the full valuation from each type of consumer, consumer surplus is

$$CS^{d}\equiv 0<CS^{n}.$$

Hence, the data collection allows the firm to price discriminate perfectly. While this yields an efficient allocation that maximizes the aggregate surplus, consumers lose as compared to the benchmark above of a world without data collection.

For future reference, it is helpful to state more formally the contract that the firm offers to the consumer from an ex ante view. In the case of data collection without consent, a contract specifies a quantity *x* ∈ {0, 1} and a price p∈R , conditional on the type revealed by the data collection:

$$x^{d}\left(v\right)\equiv 1;\;p^{d}\left(v\right)\equiv v.$$

That is, in a world with data collection but without the need of consent, the following equilibrium outcome results: the firm offers to the consumer the contract (*x* ^*d*^(.), *p* ^*d*^(.)), all consumer types accept this contract, the firm learns the consumer’s type *v* from the data collection, and obtains the price *p* ^*d*^(*v*) = *v* from a consumer with valuation *v* in exchange for the good. Note that the contract (*x* ^*d*^(.), *p* ^*d*^(.)) is not a direct mechanism, as it conditions on the true type *v* rather than the consumer’s report about it.11An alternative, equivalent implementation of the contract (*x* ^*d*^(.), *p* ^*d*^(.)) that is fully in line with the CCPA’s “right to no discrimination,” is as follows. The firm offers the good at a fixed price of 1 and gives a consumer a discount of 1 − *v* if the consumer does not-opt out of a data collection so that it reveals the consumer’s willingness to pay of *v*. Indeed, as the State of California Department of Justice explains on its website: “Businesses can also offer you promotions, discounts and other deals in exchange for collecting, keeping, or selling your personal information. But they can only do this if the financial incentive offered is reasonably related to the value of your personal information. If you ask a business to delete or stop selling your personal information, you may not be able to continue participating in the special deals they offer in exchange for personal information. If you are not sure how your request may affect your participation in a special offer, ask the business.”


### 2.4 Data Collection With Consent

Next consider a digital world with data collection but now the firm, first, has to ask for the consumer’s consent in order to collect and use the data. In case the consumer does not consent, the firm cannot learn the consumer’s valuation. In case the consumer consents, the firm learns the consumer’s actual valuation.

Asking for consent formally means that the firm’s contract, (*x* ^*c*^(.), *p* ^*c*^(.)), now also conditions the quantity *x* and the price *p* on the consumer’s consent. If the consumer gives consent, the contract further conditions (*x*, *p*) on the true type as revealed by the data collection, whereas without consent it cannot.

Clearly, the firm cannot do better than without consent. To determine an optimal contract, it therefore suffices to show that also with consent, the firm can obtain the payoff Π^*d*^.

It is immediate that the firm can indeed do so by the following “service-only-on-consent” contract:

$$x^{c}\left(C,v\right)\equiv \left\{\begin{array}{cc} 1\; & \text{if }C=1\\ 0\; & \text{otherwise} \end{array}\right.\;p^{c}\left(C,v\right)\equiv \left\{\begin{array}{cc} v\; & \text{if }C=1\\ 0\; & \text{otherwise,} \end{array}\right.$$

where *C* = 1 represents the consumer giving consent, and *C* = 0 for refusing it.

The contracting game of data collection with consent has the following (unique) equilibrium outcome: the firm offers to the consumer the contract (*x* ^*c*^(.), *p* ^*c*^(.)), all consumer types accept the contract and consent, the firm learns the consumer’s type *v* from the data collection, and obtains the price *p* = *v* from consumer type *v* in exchange for the good.12Because this offer extracts the consumer’s surplus completely, it is also optimal given this offer not to give consent. However, declining the offer cannot be part of an equilibrium, because this would lead to 0 profits to the firm, implying that the firm does strictly better with a “service-only-on-consent” offer with a *p*(*v*) = *v* − *ɛ* for *ɛ* > 0 small, as for this offer it is strictly optimal for the consumer to give consent, yielding the firm the strictly higher payoff Π^*d*^ − *ɛ* > 0. Yet, any “service-only-on-consent” offer with a *p*(*v*) = *v* − *ɛ* can also not be an equilibrium, because a “service-only-on-consent” offer with a *p*(*v*) = *v* − *ɛ*/2 yields the firm strictly more. Hence, in any equilibrium outcome of data collection game with consent, the firm obtains the perfect price discrimination payoff Π^*d*^ and consumer surplus is *CS* ^*d*^ = 0.


Hence, a simple “service-only-on-consent” clause in its offer enables the firm to bypass the consent regulation completely. “Service-only-on-consent” clauses are common practice in many industries. For instance, if a customer applies for credit at a bank, the bank will only want to provide the credit if the consumer has a decent credit score. However, in order to obtain the customer’s credit report from a credit score agency, the bank needs the customer’s explicit consent for pulling his score. To ensure this consent, banks commit to refuse the credit request if the customer refuses to give the consent.13As discussed in the introduction, the European GDPR addresses such conditioning explicitly in Article 7.4, taking issue with conditioning the provision of a service “on consent to the processing of personal data that is not necessary for the performance of that contract”.


Yet, the firm can circumvent the consent regulation also if it is forced to service the consumer upon refusing consent; it commits to a service at an excessively high price, e.g., *p* = 10 > 1, whenever the consumer refuses consent. For instance, the contract

$${\hat{x}}^{c}\left(C,v\right)=1;\;{\hat{p}}^{c}\left(C,v\right)=\left\{\begin{array}{cc} v\; & \text{if }C=1\\ 10\; & \text{otherwise.} \end{array}\right.$$

also yields the firm the payoff Π^*d*^, and consumers a surplus of *CS* ^*d*^ = 0

The scheme works because consumers know that upon refusal they are faced with such a bad option that it is better to consent to the data collection. In practise, there are many other ways for the firm to make the data refusal option unpalatable to the consumer. For instance letting the consumer know that upon refusing data collection, the online service is degraded to such a degree that purchasing the product is such a hassle that its purchase is no longer worthwhile.

### Data Collection With Consent and Price-Cap $\bar{p}$

2.5

Next, I confirm two further claims of the introduction. First, with the possibility of data collection, it is indeed not optimal for the firm to offer the same terms to the consumer as the ones that obtain without the possibility of data collection. Second, if the regulation would induce the firm to offer these same terms, then the regulation does guarantee that consumers do not lose from the possibility of data collection. Hence, extending consent regulation by a regulation of the firm’s selling price when the consumer refuses consent, solves the identified problems. Such extended regulation does protect consumers against the harms of data collection, while allowing the firm to realize the potential efficiency gain of data collection.

To see this, let the extended regulation set a price-cap of $\bar{p}=p^{n}$ as specified in (1) on the price that the firm can charge consumers who do not consent to data collection. Given the price-cap, the firm can obtain at most *p* ^*n*^ from a consumer with value *v* > *p* ^*n*^, implying an upper bound on profits of

$$\left(1-F\left(p^{n}\right)\right)p^{n}+\int_{0}^{p^{n}}vdF\left(v\right).$$

The firm can obtain this upper bound by the following contract:

$$x^{e}\left(C,v\right)\equiv 1;\;p^{e}\left(C,v\right)\equiv \left\{\begin{array}{cc} v\; & \text{if }C=1\\ p^{n}\; & \text{otherwise.} \end{array}\right.$$

That is, if a consumer type *v* consents to the data collection, then the firm learns her type from the data collection and charges her a personalized price of *p* = *v*. If the consumer does not consent, the consumer can buy the good at a price *p* ^*n*^, corresponding to the price-cap as set by the extended regulation.

The contract offer (*x* ^*e*^(.), *p* ^*e*^(.)) satisfies the extended regulation and supports an equilibrium in which only consumers with a valuation *v* ≤ *p* ^*n*^ consent to the data collection and having them subsequently buy the good at their valuation. Consumers with a valuation *v* > *p* ^*n*^ do not consent to the data collection and buy the good at the price *p* ^*n*^.

Hence, the extended regulation yields an equilibrium allocation that is efficient as all consumers buy, but no consumer is worse off than in the benchmark with no data collection. At the same time, the firm strictly benefits from the data collection as it enables the firm to sell the good also profitably to low value consumers. As compared to a world with no data collection, the extended regulation leads to a Pareto improvement. All the economic benefits from data collection are realized without making anyone worse off.

While the extended regulation with a price cap of $\bar{p}=p^{n}$ as constructed above solves the problem optimally, it requires regulators to be able to derive the cap *p* ^*n*^. However, regulators often lack the detailed information required for this (e.g., knowing the exact distribution of valuations *F*(*θ*)).

This leads to the natural question how sensitive the ability to obtain Pareto improvements are to mistakes in the price cap level $\bar{p}$ . Note that if the price cap, $\bar{p}$ , exceeds *p* ^*n*^, then consumers with a valuation *v* in the interval $\left[p^{n},\bar{p}\right]$ are hurt from digitalization, as they end up paying their valuation rather than the lower price *p* ^*n*^. If the price cap $\bar{p}$ lies below *p* ^*n*^, then no consumer is worse off under digitalization, while all consumer with a valuation exceeding $\bar{p}$ strictly benefit. To the firm the difference in profits is

$$\Delta \Pi \left(\bar{p}\right)=\int_{0}^{\bar{p}}vdF\left(v\right)+\left(1-F\left(\bar{p}\right)\right)\bar{p}-\Pi^{n},$$

which is strictly positive for $\bar{p}$ close enough to *p* ^*n*^. Hence, for a range of price caps $\bar{p}\in \left[\underline{\bar{p}},p^{n}\right]$ , the data-collection with a price cap $\bar{p}$ yields a Pareto improvement, where the lower bound $\underline{\bar{p}}$ satisfies $\Delta \Pi \left(\underline{\bar{p}}\right)=0$ .14If the regulator’s benchmark would be one of pure efficiency rather than Pareto improvements vis-a-vis the benchmark of no data collection, then a price cap of zero would be optimal. It implies that price equals marginal costs so that it leads to first best efficiency, implying that it would also be optimal in the case without any data collection.


### 2.6 Data Collection with Consent and Reasonable Offers

Given that upward mistakes in setting the right price cap lead to consumers being hurt by data-collection, one may ask whether more practical, less informationally demanding forms of regulation could achieve the same outcome as by setting an optimal price cap $\bar{p}\in \left[\underline{\bar{p}},p^{n}\right]$ . To address this question, note that the contracts (*x* ^*c*^(.), *p* ^*c*^(.)) and $\left({\hat{x}}^{c}\left(.\right),{\hat{p}}^{c}\left(.\right)\right)$ enable the firm to circumvent the regulation by making the outcome without consent so unattractive to consumers that they voluntarily consent to the data collection. Hence, the driving force behind these offers is the threat of a bad outcome to consumers.

The contracts (*x* ^*c*^(.), *p* ^*c*^(.)) and $\left({\hat{x}}^{c}\left(.\right),{\hat{p}}^{c}\left(.\right)\right)$ makes these threats credible by exploiting the commitment that current consent regulation allows explicitly. In particular, the contract (*x* ^*c*^(.), *p* ^*c*^(.)) commits to not serving a dissenting consumer at all, while $\left(\hat{x},\hat{p}\right)$ commits to serving a dissenting consumer at a prohibitively high price of *p* = 10. Both offers are however unreasonable in the sense that they are not sequentially rational; they are suboptimal for any belief **E**{*v*} ∈ [0, 1] about the consumer’s expected valuation that the firm may have when actually being confronted with a dissenting consumer.

This raises the question whether the identified loophole of consent regulation can be resolved by extending it by prohibiting sellers to make unreasonable offers which are not sequentially rational.15This idea is akin to demanding sequentially rationality as part of the equilibrium concept in extensive form games, which, as is well-known, generally severely limits equilibrium outcomes. As discussed in the introduction, the CCPA, in fact, demands such reasonability in CIV 1798.125.2. I next argue that if we take a reasonable offer as one that is sequentially rational, the answer is negative.16See Jaeger (2023) for different approaches towards operationalizing the concept of “reasonableness” in law.


Indeed, changing the price in the contract $\left({\hat{x}}^{c},{\hat{p}}^{c}\right)$ in case of no consent (*C* = 0) from 10 to 1, we obtain a contract that induces an equilibrium in which all consumers with a valuation *v* ∈ [0, 1) consent to the data collection, whereas a consumer with valuation *v* = 1 dissents. The price *p* = 1 is therefore indeed sequentially rational, because the seller correctly anticipates that a dissenting consumer has the valuation *v* = 1 so that it is indeed sequential rational to set the price *p* = 1. Note that the scheme is also reasonable in the full sense of the CPPA because it is perfectly relates “to the value provided to the business by the consumer’s data”.

Hence, requiring the firm to make only sequentially rational offers does not prevent the firm from circumventing the regulation. Note that this result is general because it effectively follows from the fact that data collection makes the consumer’s private information verifiable and that private information that is verifiable typically unravels (e.g. Grossman 1981, Milgrom 1981). Indeed, the only price that is sequentially rational is the price *p* = 1 to dissenting consumers. In particular, the price *p* ^*n*^ is not sequentially rational because at this price it would be optimal for a consumer with value *v* to dissent if and only if *v* > *p* ^*n*^. Hence, after seeing a refusal, the seller rationally concludes that the consumer must have a value of at least *p* ^*n*^ but then the price *p* ^*n*^ is not optimal.

## 3 Conclusions

This paper demonstrates that, due to firms’ strategic use of “dissent offers”, current consumer consent regulations, while seemingly empowering, are insufficient to protect consumers from exploitation. The analysis, based on a stylized but canonical model of monopolistic screening, where a monopoly uses consumer data for personalized pricing, reveals that firms can design unattractive dissent offers that effectively coerce consumers into consenting to data collection, undermining the intended control over their data.

The analysis yields three key insights for privacy regulation. First, simple consent regulation alone is ineffective because firms can commit to dissent offers that leave consumers no meaningful choice. Second, effective protection requires explicit regulation of dissent offers, with an optimally set price cap generating a Pareto improvement over the pre-data-collection world. Third, attempts to require “reasonable” offers defined as sequentially rational are also ineffective due to the unraveling properties of verifiable information.

While these findings are robust within the theoretical framework presented, several limitations and extensions warrant discussion.

In assuming that firms can make arbitrary dissent offers, the paper takes an extreme position. In real life markets, firms face many frictions such as competition, reputational concerns, and consumer backlash that may constrain such offers. Incorporating these real-world frictions would enhance the model’s applicability. For instance, in markets with greater competition, firms might be limited in their ability to impose unattractive dissent offers due to the risk of losing customers to competitors.

The model’s assumption of costless and perfect data collection illustrates an extreme scenario. While it serves as a useful benchmark, exploring scenarios with costly or imperfect data collection would provide a more realistic analysis. In such situations, consumer surplus might not be driven to zero, and the firm’s ability to extract surplus would be limited. However, popular press often cites examples, indicating that data-collection allows firms to know us better than we know ourselves.17E.g., Wired (2018) reports that Amazon will soon know when you need lightbulbs right before they burn out and YouTube knows how to keep you staring at the screen long past when it is in your interest to stop (see https://www.wired.com/story/artificial-intelligence-yuval-noah-harari-tristan-harris/, last retrieved 26.2.2025). As a concrete example of how data analysis could potentially “know us better than we know ourselves,” Harari (2016) argues that by analyzing patterns in how someone browses websites and engages with content, algorithms can identify a person’s sexual orientation well before they have come to that realization themselves. Hence, while extreme, the model’s assumption that firms learn the consumer’s private information perfectly may actually be understating the firm’s abilities from data-collection.

In addition, many online services do not involve direct monetary prices, which affects how firms can implement discriminatory offers. Instead of price, firms might adjust content or service quality, which could be costly to implement. Analyzing these alternative forms of discrimination would broaden the scope of the analysis.

Moreover, the current model assumes consumers are passive recipients of offers, but in reality, consumers may strategically behave to signal they have a low valuation. Allowing for such strategic consumer behavior would add depth to the analysis.

The regulatory solution of a price cap equal to the static “non-data-collection” monopoly price theoretically solves the problem, but faces significant implementation challenges due to its informational demands. However, even imperfect price caps can generate welfare improvements. For any price cap below but close to non-data-collection price, data collection with consent regulation remains Pareto-improving relative to no data collection.

Defining reasonable offers as sequentially rational leads to outcomes where firms can still circumvent regulations. Exploring alternative definitions of reasonableness and their implications is essential for effective policy design.

By addressing these points, future research can provide a more comprehensive understanding of consumer consent regulations and their implications in the digital economy. Yet, the fundamental mechanism that the stylized model identifies – strategic design of dissent offers – likely remains also a crucial factor in more realistic scenarios of digital consumer markets.
